# Reduced expression of chemokine (C-C motif) ligand-2 (CCL2) in ovarian adenocarcinoma

**DOI:** 10.1038/sj.bjc.6602596

**Published:** 2005-05-17

**Authors:** J M Arnold, P R Huggard, M Cummings, G A Ramm, G Chenevix-Trench

**Affiliations:** 1 The Queensland Institute of Medical Research, Brisbane, Australia; 2 Department of Pathology, University of Queensland, Australia

**Keywords:** ovarian adenocarcinoma, CCL2, MCP-1, 5-aza-2′-deoxycytidine, SSCP, *in situ* hybridisation

## Abstract

Chemokine (C-C motif) ligand-2 (*CCL2*) is a chemoattractant and activator of macrophages and is a key determinant of the macrophage infiltrate into tumours. We demonstrate here that *CCL2* is expressed in normal human ovarian surface epithelium (HOSE) cells and is silenced in most ovarian cancer cell lines, and silenced or downregulated in the majority of primary ovarian adenocarcinomas. Analysis of the *CCL2* locus at 17q11.2–q12 showed loss of heterozygosity (LOH) in 70% of primary tumours, and this was significantly more common in tumours of advanced stage or grade. However, we did not detect any mutations in the *CCL2* coding sequence in 94 primary ovarian adenocarcinomas. These data support the hypothesis that *CCL2* may play a role in the pathobiology of ovarian cancers, but additional studies will be required to evaluate this possibility.

Ovarian cancer is the leading cause of death from gynaecological neoplasias, and the sixth most common cancer among women worldwide (Parkin *et al*, 1999). The lack of symptoms during the early stages of the disease results in the majority of women presenting with advanced tumours that do not respond well to treatment. Consequently, the overall 5-year survival rate is only 30%. Most ovarian cancers, the adenocarcinomas, arise as a consequence of the accumulation of genetic or epigenetic inactivation of tumour suppressor genes and activation of oncogenes in the ovarian surface epithelium (OSE) (reviewed in Aunoble *et al*, 2000; Liu and Ganesan, 2002). Ovarian neoplasms occur in benign, low malignant potential (LMP) and malignant forms although the relationship between these forms is not clear, nor are the genetic and biological events that underlie the initiation and progression of ovarian adenocarcinomas.

Ovarian tumours may contain a significant number of infiltrating host leukocytes, and in the case of ovarian cancers, these are predominantly macrophages and T cells (Merogi *et al*, 1997; Negus *et al*, 1997). The likely stimulus for the presence of these leukocytes is the local production of chemokines. Chemokines are a family of small, secreted, proinflamatory cytokines with chemotactic activity against leukocytes (Oppenheim *et al*, 1991). In particular, chemokine (C-C motif) ligand 2 (CCL2, previously known as monocyte chemotactic protein-1, MCP-1), which is a potent chemoattractant for macrophages (Sica *et al*, 1990), and also T cells (Taub *et al*, 1995), has been found to be expressed in ovarian tumours (Negus *et al*, 1995; Burke *et al*, 1996) and appears to be an important determinant of the leukocyte infiltrate into tumours (Zacchariae *et al*, 1990; Rollins, 1991; Bottazzi *et al*, 1992; Negus *et al*, 1997; Zhang *et al*, 1997).

However, the role of these tumour-associated leukocytes remains unclear (Mantovani *et al*, 1992; Mantovani, 1994; O'Sullivan and Lewis, 1994). It is known that they can mount an immune response against malignant cells and kill them (Parmiani *et al*, 1990; Rollins and Sunday, 1991; Colombo *et al*, 1992; Peoples *et al*, 1995; Goedegebuure *et al*, 1997) and, indeed, the presence of intratumoural T cells in ovarian cancer patients has been shown to be significantly associated with increased progression-free and overall survival (Zhang *et al*, 2003). On the other hand, macrophages can produce factors that help tumour growth and/or vascularisation through paracrine loops (Malik and Balkwill, 1991; Mantovani *et al*, 1992). The particular role of these cells in a given case depends on factors such as the activation state of the macrophages and the intrinsic properties of the tumour cell, and is known as the macrophage balance (Mantovani, 1994).

Using cDNA array screening of normal OSE cells and ovarian cancer cell lines, we previously identified *CCL2* as a gene whose expression is downregulated or lost in ovarian cancer cell lines (Arnold *et al*, 2001a). In this report, we describe the characterisation of *CCL2* expression in a larger panel of ovarian cancer cell lines and primary tumours and mutation analysis of the *CCL2* gene in primary ovarian adenocarcinomas.

## MATERIALS AND METHODS

### Cell lines, OSE cultures and primary tumours

Human ovarian surface epithelial cell lines (HOSE) 17.1 and 1.1, immortalised with a replication-defective retroviral construct expressing human papillomavirus oncogenes (Tsao *et al*, 1995), were maintained in a medium composed of 1 : 1 M199 : MCDB105 with 10% FCS. The HEY (Buick *et al*, 1985) and OVCAR4, OVCAR5 and OVCAR8 (kind gift from Dr E Marshall, Auckland Cancer Society) and OVHS-1 (kind gift of Dr N Ahmed, Royal Women's Hospital, Melbourne) ovarian cancer cell lines were maintained in MEM alpha medium with 10% FCS. OAW 42 (Wilson, 1984), OAW 28+53 (Wilson *et al*, 1987), PEO1 and PEO4 (Wolf *et al*, 1987), PEO14 (Langdon *et al*, 1988), JAM (Ward *et al*, 1987), SKOV3 (Fogh and Trempe, 1975), COLO316 (Woods *et al*, 1979), CAOV3 (Wong *et al*, 1999), OVCAR-3 (Hamilton *et al*, 1983), A2780 (established by Dr S Aaronson, National Cancer Institute), CI80135 and 27/87 (established by T Hurst, Obstetrics and Gynecology, Royal Brisbane Hospital) were all maintained in RPMI 1640 with 10% FCS. OVCA420, OVCA432, OVCA433 and DOV13 (kind gifts from Dr S Mok, Laboratory of Gynecologic Oncology, Brigham and Women's hospital, Boston, MA, USA) were maintained in a mixture of 1 : 1 MCDB105 and M199 with 10% FCS.

Cells were harvested for RNA at about 80% confluence. The cell lines OAW42, PEO1, PEO4, PEO14, JAM, SKOV3 and COLO316 were derived from serous tumours, and 27/87 was from an endometrioid tumour. The histological origin of the remaining cell lines is not available.

Uncultured HOSE cells were obtained by scraping stromal cells away from epithelial sheets. Their distinctive cellular morphology was used to confirm that the cells were epithelial. RNA was extracted directly from these peeled epithelial cells without culturing.

Ovarian adenocarcinomas were obtained from 97 patients undergoing surgery. There were 68 serous tumours, 13 endometrioid tumours, nine mucinous tumours, five clear-cell tumours and two tumours of mixed histology. The series included one benign and 10 LMP tumours as well as 86 malignant tumours. All patients were staged at laparotomy, in accordance with the recommendations of the International Federation of Gynaecology and Obstetrics (FIGO). Of the LMP tumours, five were FIGO stage 1 and four were stage 3 (one was of unknown stage) and of the malignant tumours, seven were FIGO stage 1, five stage 2, 66 stage 3 and eight stage 4. Constitutional DNA was available in all cases from peripheral blood.

### Isolation of DNA and RNA

Tumour tissue was dissected free from necrotic and connective tissue, and mechanically dispersed prior to collagenase treatment (0.1 mg ml^−1^ in Hanks balanced salt solution). Erythrocytes and necrotic cells were removed with ficoll-paque, and genomic DNA was extracted by the salting-out method as described elsewhere (Miller *et al*, 1988). Total RNA was extracted from fresh primary tumours and subconfluent cultured cell lines using Tri-reagent (Sigma, Castle Hill, NSW), following the manufacturers' instructions. PolyA+ RNA was prepared from total RNA using Dynabeads (Dynal, Carlton South, Vic), according to the recommended protocol.

### Northern blot analysis

RNA was denatured and electrophoresed on a formaldehyde–agarose gel and transferred onto a nylon membrane by capillary blotting overnight according to standard protocols (Sambrook and Russell, 2001). The RNA was then fixed to the membrane by UV irradiation. Probes were prepared from RT–PCR products by random priming and hybridisation was carried out for 2 h in ExpressHyb solution (Clontech, Palo Alto, CA, USA) at 65°C and then the membrane was subjected to standard washing procedures and autoradiography.

### Semiquantitative RT–PCR

cDNA synthesis was carried out with the Superscript II reverse transcriptase on 1 *μ*g of total RNA primed with random hexamers. RT–PCR was performed with primers for both *CCL2* and *β*-actin in a multiplex reaction in a total volume of 20 *μ*l incorporating ^33^P-labelled dATP using standard PCR cycling conditions with annealing at 60°C. The reaction was stopped and 5 *μ*l of product removed at cycles 24, 28, 32 and 36 to ensure linear amplification and the products were run on a denaturing acrylamide gel prior to autoradiography. The primer sequences for both *CCL2* (CTAAGCTTCCAGCATGAAAGTCTCTGG and GTGAGTGTTCAAGTCTTCG) and *β-actin* (CGTGACATTAAGGAGAAGCTGTGC and CTCAGGAGGAGCAATGATCTTGAT) spanned at least one intron to ensure quantitation was assessed only on amplified cDNA.

### mRNA *in situ* hybridisation (ISH)

The *CCL2* full-length cDNA was subcloned into pGEM-T vector (Promega, Annandale, NSW). Digoxigenin-labelled riboprobes for sense and antisense orientations were produced by *in vitro* transcription with SP6 and T7 RNA polymerases (Roche, Mannheim, Germany) as previously described (Ramm *et al*, 1998) and then subjected to alkaline hydrolysis. Sections were deparaffinised with xylene, rehydrated in an alcohol gradient and subjected to hydrochloric acid (0.2 mol l^−1^), as previously described (Rex and Scotting, 1994). Sections were permeabilised with 20 *μ*g ml^−1^ proteinase K for 15 min at 37°C and fixed in 4% paraformaldehyde for 20 min at room temperature. Prehybridisation (4 × SSC/50% (v v^−1^) formamide) was performed at 42°C for 4 h followed by hybridisation for 18 h at 42°C with digoxigenin-labelled riboprobe at a concentration of 1 *μ*g ml^−1^ in a solution of 40% (v v^−1^) formamide, 10% (w v^−1^) dextran sulphate, 1 × Denhardt's solution, 4 × SSC, 10 mM DTT and 1 mg ml^−1^ yeast tRNA.

Sections were washed to remove unbound probe and then incubated with alkaline phosphatase-conjugated antidigoxigenin polyclonal sera (1 : 100) (Roche, Mannheim, Germany) at room temperature for 2 h. Unbound antibody was removed by washing, followed by visualisation with nitroblue tetrazolium chloride/5-bromo-4-chloro-3-indolyl phosphate (Roche, Mannheim, Germany) in the dark at room temperature for 18 h. Unbound complex was removed by washing and sections were counterstained with eosin. Sections were visualised with a Nikon Eclipse E800 microscope and photographed with a Nikon DXM1200F digital camera.

### Loss of heterozygosity (LOH) analyses

Loss of heterozygosity was assessed at 17q11.2–q12 with the D17S1293 and D17S933 microsatellite markers, which are located 0.02 Mb proximal and 0.61 Mb distal to *CCL2* at 32.73 Mb (www.ncbi.nlm.nih.gov/). PCR amplification was carried out for 35 cycles in the presence of ^33^P-labelled dATP and PCR products were analysed on a denaturing polyacrylamide gel. Loss of heterozygosity was scored conservatively as a clear reduction in the intensity of one allele (>70%) by two independent observers, one of whom was blind with respect to the sample identity.

### Single-strand conformation polymorphism analysis

Primers were designed to intronic regions to amplify each of the three coding exons of the human *CCL2* gene. A total of 94 primary ovarian tumours obtained prior to chemotherapy were screened and constitutional DNA from blood was available for all. DNA samples were amplified in the presence of ^33^P-labelled dATP using standard PCR cycling conditions with annealing at 60°C, denatured at 95°C for 5 min and then electrophoresed on 0.5 × MDE (FMC Biotech, Rockland, Maine) gel overnight at room temperature.

The primers used for SSCP and product sizes were: exon 1 CAATAAGAGGCAGAGACAGCAGCCAG and GTTAAAGCAAGACTGTGGGTACCACG (273 bp); exon 2 GCTCTTTCTCTTCTCCTGCCTGC and GAGGCTTGTCCCTTGCTCCACAAGG (300 bp); exon 3 CCTCCTAGTCTCCATGGCAGCTCGC and ACAGGGTGTCTGGGGAAAGCTAGG (253 bp). PCR products amplified from these primers span all coding exons of the *CCL2* gene and include sequences 72 bp upstream of the start codon, 44 bp downstream of the stop codon and at least 40 bp of flanking intronic sequences exclusive of the primers.

### 5-aza-2′-deoxycytidine treatment of ovarian adenocarcinoma cell lines

Cell lines were plated at 20–30% confluence and treated 24 h later (day 0) with 0, 0.5 or 2.0 *μ*M 5-aza-2′-deoxycytidine. Fresh media containing the same concentration of 5-aza-2′-deoxycytidine was added on day 2 and cells were harvested for RNA extraction on day 5.

## RESULTS

### *CCL2* expression analysis in ovarian adenocarcinoma cell lines

We previously conducted a screen for genes aberrantly expressed in three ovarian adenocarcinoma cell lines compared to an immortalised HOSE using a human cDNA array containing 588 known genes (Arnold *et al*, 2001a). This showed that expression of *CCL2* is greatly reduced in ovarian cancer cell lines. This finding was confirmed using semiquantitative RT–PCR analysis which showed that *CCL2* was expressed in both of the HOSE cell lines tested, while three out of four ovarian cancer cell lines did not express and one out of four had reduced levels of expression (Arnold *et al*, 2001a).

**Figure 1:**
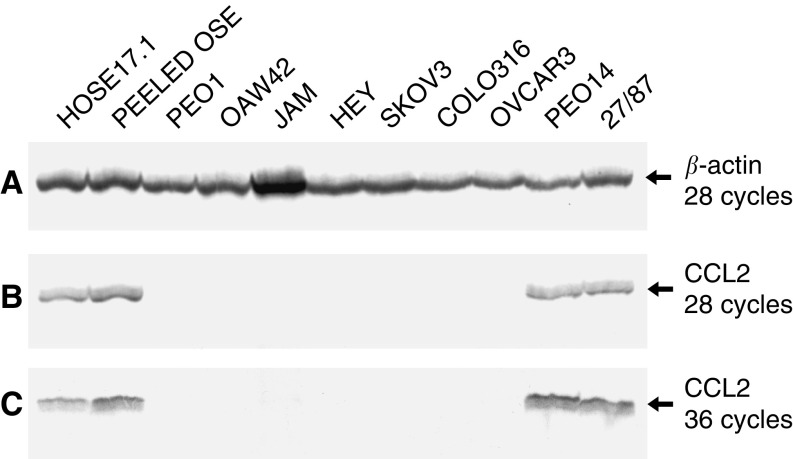
Analysis of *CCL2* expression in immortalised HOSE cells, peeled (uncultured) OSE cells and ovarian adenocarcinoma cell lines by RT–PCR. Multiplex PCR was carried out for CCL2 and *β*-actin on cDNA, run on a polyacrylamide gel, and exposed to film. (**A**) PCR products for *β*-actin after 28 cycles of amplification. (**B**) PCR products for CCL2 after 28 cycles of amplification. (**C**) PCR products for CCL2 after 36 cycles of amplification.

To further extend these results, semiquantitative RT–PCR was repeated to include peeled (uncultured) normal OSE cells as well as the HOSE17.1 cell line and a broader panel of ovarian cancer cell lines. Expression of *CCL2* was detected at similar levels in the HOSE17.1 cell line, the uncultured normal OSE cells, and the PEO14 and 27/87 ovarian cancer cell lines (Figure 1A, B). No *CCL2* expression was detected in the remaining seven ovarian adenocarcinoma cell lines, even after 36 rounds of PCR amplification (Figure 1C).

**Figure 2:**
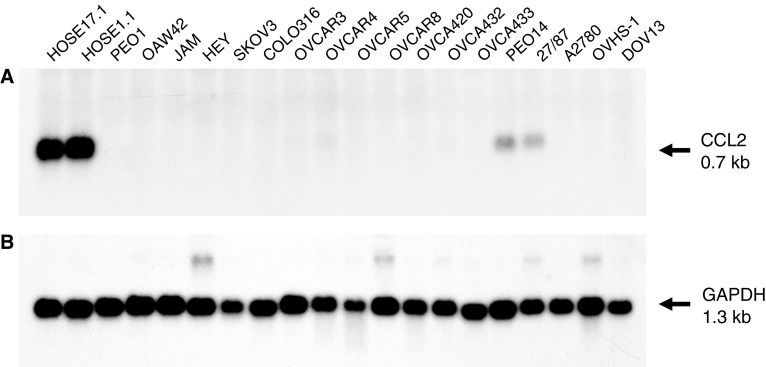
Analysis of CCL2 expression in HOSE and ovarian adenocarcinoma cell lines by Northern blotting. Each lane represents 2 *μ*g of polyA+ RNA. (**A**) Hybridisation with the *CCL2* probe. (**B**) Hybridisation with the glyceraldehyde-3-phosphate dehydrogenase *(GAPDH)* probe.

The expression of *CCL2* mRNA was next examined in a larger panel of HOSE and ovarian cancer cell lines by Northern blot analysis (Figure 2). Strong expression was detected in the HOSE17.1 and HOSE1.1 cell lines, while much weaker expression was found in the PEO14 and 27/87 cell lines. No expression was detected in the remaining 16 ovarian adenocarcinoma cell lines.

### CCL2 expression analysis in primary ovarian adenocarcinomas

**Figure 3:**
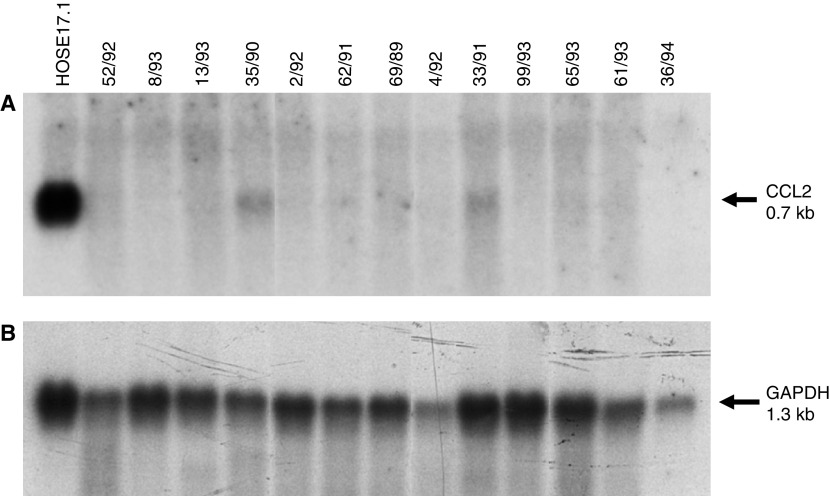
Analysis of CCL2 expression in ovarian primary adenocarcinomas by Northern blotting. Lane 1, HOSE17.1 cell line; lanes 2–9, ovarian serous adenocarcinomas; lanes 10–13 ovarian endometrioid adenocarcinomas; lane 14 mucinous ovarian adenocarcinoma. Each lane represents 5 *μ*g of total RNA. (**A**) Hybridisation with the *CCL2* probe. (**B**) Hybridisation with the glyceraldehyde-3-phosphate dehydrogenase *(GAPDH)* probe.

Having demonstrated that *CCL2* is strongly expressed in cultured and uncultured HOSE cells, and that expression is lost or reduced in the vast majority of ovarian cancer cell lines, we next investigated its expression in primary ovarian tumours. Northern blot analysis of the HOSE17.1 cell line and 13 primary adenocarcinomas revealed greatly reduced levels of expression in two primary tumours (35 out of 90 and 33 out of 91) compared to the HOSE17.1 cells, while no expression was detected in the remaining 11 primary tumours (Figure 3).

**Figure 4:**
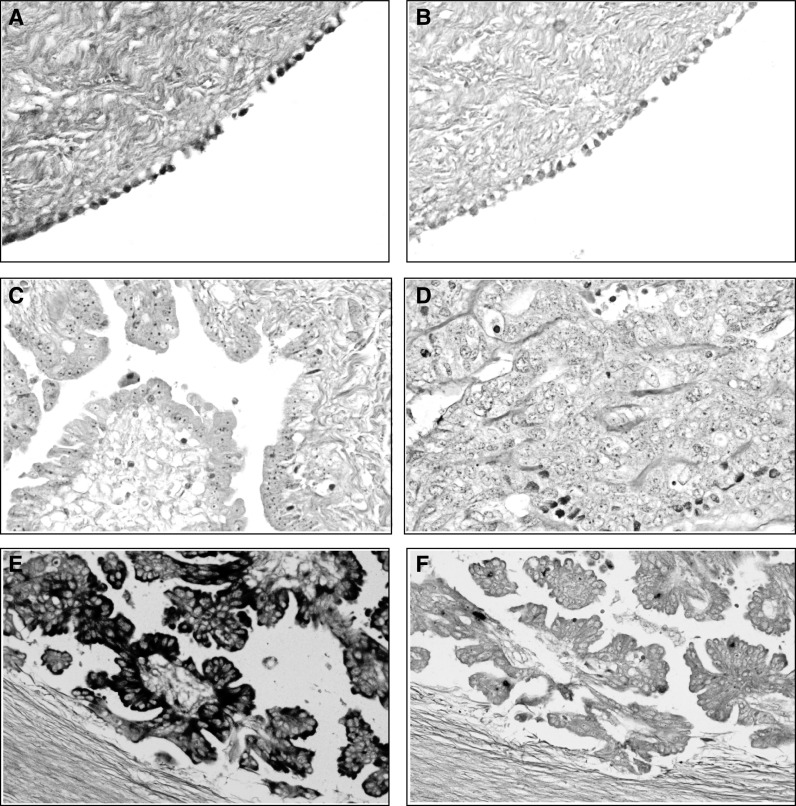
*In situ* hybridisation of CCL2 in ovarian tumours and normal ovaries. Normal ovary hybridised with (**A**) antisense *CCL2* probe (× 40) and (**B**) sense control probe (× 40) showing staining in the majority of surface epithelial cells. Ovarian adenocarcinomas hybridised with antisense *CCL2* probe (**C, D, E**) or sense control probe (**F**). Tumours 97/93 (**C**, × 40) and 99/93 (**D**, × 40) show very little staining for *CCL2*. Ovarian adenocarcinoma 52/92 hybridised with (**E**) antisense *CCL2* probe (× 40) and (**F**) sense control probe (× 20). The majority of 52/92 tumour cells in this field show *CCL2* expression.

To further investigate the extent and localisation of *CCL2* expression in primary ovarian tumours, we used mRNA ISH to analyse two normal ovaries and 13 primary adenocarcinomas. This series included nine adenocarcinomas that we had previously investigated by Northern blot. A pathologist (MC) scored the percent of expressing cells into categories. No *CCL2* expression was detected in the stroma of any of the tumours. Four tumours expressed *CCL2* in 0–5% of epithelial cells, six tumours expressed *CCL2* in 6–25% of epithelial cells, while only three tumours expressed *CCL2* in greater than 25% of the epithelial cells. In the normal ovaries, *CCL2* expression was detected in 60–80% of the surface epithelial cells and also epithelial cells lining invaginations and inclusion cysts. Representative photomicrographs of *CCL2* staining in a normal ovary and three tumours are shown in Figure 4.

There were nine tumours, which were analysed by both ISH and Northern blot. Of the seven tumours in which expression was not detected by Northern blot, six had less than 25% epithelial cells expressing, while one (61 out of 93) had 26–50%, and of the two which did show expression on the Northern blot, one tumour was scored as having 50–75% expression while the other was scored as 6–25%. The lack of correlation between these two methods in tumour 61 out of 93 is probably due to tumour heterogeneity, since the sections used for ISH were not adjacent to the tissue used to isolate RNA.

### 5-azacytidine treatment of cell lines

**Figure 5:**
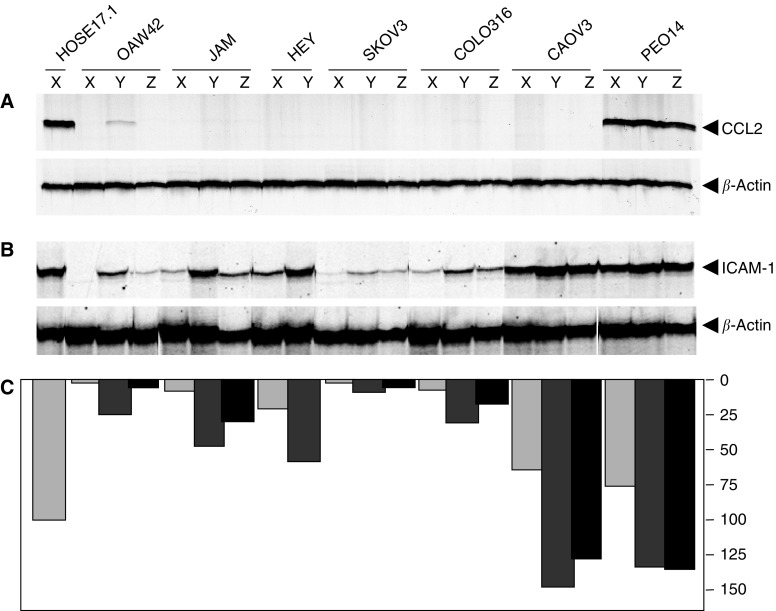
Analysis of CCL2 expression in ovarian cancer cell lines following treatment with 5-azacytidine. Cell lines were treated with 0 (X), 0.5 (Y) or 2.0 (Z) *μ*M 5-azacytidine and RNA harvested 5 days later. (**A**) Multiplex PCR carried out with primers for *β-*actin and *CCL2* for 28 cycles. (**B**) Multiplex PCR carried out with primers for *β-*actin and *ICAM-1* as previously described (Arnold *et al*, 2001b). (**C**) Quantitation of intensity of bands for *ICAM-1* normalised to *β-*actin.

To test whether the silencing of *CCL2* expression in the ovarian cancer cell lines is related to hyper-methylation of CpG dinucleotides, we treated cell lines with 0, 0.5 or 2.0 *μ*M of the methyltransferase inhibitor 5-azacytidine. RNA was extracted from the cultures after 5 days and the level of expression of *CCL2* was examined by semiquantitative RT–PCR (Figure 5). Consistent with previous results, the untreated controls showed no *CCL2* expression in the OAW42, JAM, HEY, SKOV3, COLO316 or CAOV3 cell lines, while strong expression was observed in the PEO14 cells. There was a slight induction of *CCL2* expression in the OAW42 cell line treated with 0.5 *μ*M 5-azacytidine, but no induction of expression in any of the other six cell lines (Figure 5A). Previous analysis of the same RNA samples for *ICAM-1* expression (Arnold *et al*, 2001b) showed induction of *ICAM-1* in cell lines treated with 5-azacytidine, demonstrating that the 5-azacytidine treatment was successful (Figure 5B, C).

### Loss of heterozygosity analysis at 17q11.2–q12

Loss of heterozygosity analysis was carried out on 41 ovarian adenocarcinomas with the D17S1293 and D17S933 microsatellite markers, which are located 0.02 Mb proximal and 0.61 Mb distal to *CCL2* at 32.73 Mb (www.ncbi.nlm.nih.gov/). Loss of heterozygosity was detected in 19 out of 27 (70%) informative cases for D17S1293 and 19 out of 26 (73%) informative cases for D17S933. Overall, LOH was detected in at least one marker in 22 out of 32 (69%) of informative cases. The LOH was significantly more common in Stage 3–4 tumours than Stage 1–2 tumours (*P*=0.03, Fisher's exact test), and tended to occur in tumours of advanced grade (*P*=0.058, Mantel–Haenszel *χ*^2^ test for trend). Of the informative tumours that did not express *CCL2* by Northern blot, six had LOH and one had no loss, while one tumour that did express *CCL2* also had LOH. Similarly, of the informative tumours that had 0–25% cells positive for *CCL2* by ISH, four had LOH and two had no loss, while there were two tumours with greater than 26% of cells positive that both had LOH.

### Mutation analysis of the *CCL2* gene

Single-strand conformation polymorphism (SSCP) analysis was carried out on all three exons of the *CCL2* gene, including sequences 72 bp upstream of the start codon and 44 bp downstream of the stop codon and at least 40 bp of flanking intronic sequences surrounding each exon. There were no aberrant bands detected in the PCR of any exon of *CCL2* in the panel of 94 primary ovarian adenocarcinomas tested.

## DISCUSSION

We first detected *CCL2* as a gene whose expression is downregulated in ovarian adenocarcinoma cell lines relative to OSE cells by cDNA array (Arnold *et al*, 2001a). Further analysis of *CCL2* expression presented here confirms that it is strongly expressed in peeled (uncultured) normal HOSE cells and immortalised cell lines derived from OSE cells. Expression is silenced in most ovarian cancer cell lines and silenced or downregulated in the majority of ovarian primary adenocarcinomas. This suggests that a selective advantage may result from downregulation of *CCL2* expression, consistent with a role of *CCL2* in the pathobiology of ovarian cancer.

A previous study of *CCL2* expression in seven ovarian cancer cell lines by RT–PCR and ELISA, found the highest levels in PEO14 and HL60, with lower levels in OVCAR3, PEO1 and PEO4 and barely detectable expression in SKOV3 and PEA2 cells (Negus *et al*, 1995). This is consistent with our results in which we also found high levels of *CCL2* mRNA in PEO14 and no expression in most other cell lines. In primary tumours, mRNA ISH showed expression of *CCL2* in an average of 1.4% of cells in 23 out of 26 primary ovarian tumours, and ELISA detected CCL2 protein in ascites from ovarian cancer patients with a mean level of 4.28 ng ml^−1^ (Negus *et al*, 1995). This is also consistent with our Northern blot results, although we detected higher levels of expression than Negus *et al* by ISH. The same study did not detect *CCL2* mRNA in the normal ovarian mesothelium (OSE), which was examined in only one case. In contrast, we have demonstrated strong expression in both cultured immortalised and uncultured OSE cells by RT–PCR, Northern blotting and ISH. This is an important new finding and demonstrates that *CCL2* expression is silenced or downregulated in the majority of ovarian cell lines and primary tumours.

Expression of *CCL2* has also been found to be decreased in prostate adenocarcinoma compared to benign prostate hyperplasia (Mazzucchelli *et al*, 1996) and cervical carcinomas compared to normal or hyperplastic squamous epithelium (Riethdorf *et al*, 1998; Kleine-Lowinski *et al*, 1999). In contrast, *CCL2* expression has been found to be increased in malignant glioma (Kuratsu *et al*, 1993; Takeshima *et al*, 1994), primary and metastatic melanoma (Graves *et al*, 1992) and glioblastomas and astrocytomas (Desbaillets *et al*, 1994) compared to corresponding normal tissues. *CCL2* expression has also been found to be increased in paclitaxel resistant cell lines and in the serum of ovarian cancer patients following paclitaxel treatment (Duan *et al*, 1999; Penson *et al*, 2000). However, rather than being associated with paclitaxel resistance, it is postulated that the increased *CCL2* expression may simply reflect stress in the host (Penson *et al*, 2000).

To investigate the mechanism of downregulation of expression of *CCL2*, we treated a panel of ovarian cancer cell lines with the methyltransferase inhibitor 5-azacytidine. This resulted in a small increase in expression levels in only the OAW42 cell line, while there was no induction of expression in the other six. This suggests that hypermethylation of CpG islands near the *CCL2* gene is not the major cause of the silencing of expression. Indeed, a search of the *CCL2* genomic region from 7 kb upstream of the start codon to 2.5 kb downstream of the stop codon (GenBank Accession Number Y18933) using Cpgplot (www.angis.org.au) did not detect any CpG islands, which further supports our findings.

We next investigated whether genetic changes may be responsible for the downregulation of *CCL2* expression. There was no evidence of any mutations in the *CCL2* gene in 94 primary ovarian adenocarcinomas, demonstrating that *CCL2* is not inactivated by somatic mutations, nor is downregulation of its expression the result of mutation in the coding or adjacent flanking sequences. There are five single-nucleotide polymorphisms reported within the *CCL2* locus (http://www.ncbi.nih.gov/SNP/); however, only two of these were within the region screened for mutations and each of these had minor allele frequencies of less than 0.5% so it is not surprising that these were not detected within the 94 ovarian cancer samples we screened.

An overall LOH frequency of 69% was detected at the *CCL2* locus at 17q11.2–q12, but there was no association with loss of *CCL2* expression. Loss of heterozygosity on chromosome 17 frequently involves the whole chromosome (Shelling *et al*, 1995), but minimal regions of loss have been detected at 17p13, 17q12.2, 17q21 and 17q25.1–qter (Liu and Ganesan, 2002). Our finding of high rates of LOH at 17q11.2–q12 places this region among the most frequently deleted regions in ovarian cancer (Shelling *et al*, 1995; Liu and Ganesan, 2002). The fact that this LOH is significantly more common in late stage than early stage tumours, indicates that it is associated with progression rather than initiation. Further analysis of 17q is required to refine the minimal region of deletion at 17q11.2–q12 and to determine whether *CCL2* is the target of this loss.

Transfection with human *CCL2* has been reported to suppress the *in vivo* growth and tumorigenicity of CHO cells (Rollins and Sunday, 1991) and melanoma cells (Bottazzi *et al*, 1992), and the fusion of tumorigenic HeLa cells, which do not express *CCL2*, with normal fibroblasts, resulted in *CCL2* expressing somatic hybrids unable to form tumours in nude mice (Rosl *et al*, 1994). In contrast, no effect on tumour growth was found for human or murine *CCL2* transfected into colon adenocarcinoma cells (Hirose *et al*, 1995). We transfected the HEY and OVCAR8 ovarian cancer cell lines with *CCL2* cloned into pcDNA3.1, but were unable to develop stable clones with comparable levels of expression to the HOSE cells, and so were unable or to interpret the consequences of *in vitro* or *in vivo* assays (data not shown).

The consequences of the introduction of *CCL2* into biologically early, nontumorigenic melanoma cells were found to depend on the level of CCL2 secretion (Nesbit *et al*, 2001). Low level secretion was found to stimulate tumour growth through angiogenesis mediated by monocyte activation, whereas high levels of CCL2 attracted large numbers of monocytes/macrophages and rapid tumour destruction (Nesbit *et al*, 2001). Thus, it appears that the levels of CCL2 may determine the biological effect. The reduced levels of CCL2 found in ovarian cancers may be promoting tumour growth through various growth factors produced by monocytes attracted to the tumour, in addition to evading the destructive response of the host macrophages and T cells. This concept requires more investigation in ovarian cancer and other neoplasias.
